# Repurposing alcohol-abuse drug disulfiram for the treatment of KSHV-infected primary effusion lymphoma by activating antiviral innate immunity

**DOI:** 10.1371/journal.ppat.1012957

**Published:** 2025-03-04

**Authors:** Lijie Wang, Zhenshan Liu, Zeyu Xu, Wenjing Wang, Jinhong Yang, Junjie Zhang, Shanping He, Qiming Liang, Tingting Li

**Affiliations:** 1 Hunan International Joint Laboratory of Animal Intestinal Ecology and Health, Laboratory of Animal Nutrition and Human Health, Hunan Provincial Key Laboratory of Animal Intestinal Function and Regulation, College of Life Sciences, Hunan Normal University, Changsha, Hunan, China,; 2 Shanghai Institute of Immunology, Department of Immunology and Microbiology, Key Laboratory of Cell Differentiation and Apoptosis of Chinese Ministry of Education, Shanghai Jiao Tong University School of Medicine, Shanghai, China; University of Southern California, UNITED STATES OF AMERICA

## Abstract

Cancer remains a leading cause of global mortality, characterized by high treatment costs, and generally poor prognoses. Developing new anti-cancer drugs requires substantial investment, extended development timelines, and a high failure rate. Therefore, repurposing existing US Food and Drug Administration (FDA)-approved drugs for other diseases as potential anti-cancer therapies offers a faster and more cost-effective approach. Primary effusion lymphoma (PEL) is an aggressive B-cell malignancy linked to Kaposi’s sarcoma-associated herpesvirus (KSHV) infection. In this study, we identified that disulfiram (DSF), an FDA-approved medication for alcohol dependence, acts as a potent inhibitor of KSHV-positive PEL. DSF suppresses PEL cell proliferation by inducing apoptosis through the activation of innate antiviral immunity. Remarkably, DSF effectively impedes KSHV reactivation and virion production in both PEL and endothelial cells. Inhibition of TANK binding kinase 1 (TBK1) or interferon regulatory factor 3 (IRF3), essential activators of antiviral innate immunity, reverses DSF’s effects on PEL cell survival and KSHV reactivation. Furthermore, DSF treatment significantly hinders the initiation and progression of PEL tumors in a xenograft mouse model, with this effect was notably abolished by TBK1 depletion. Our findings highlighted DSF as a promising therapeutic agent for targeting persistent KSHV infection and treating PEL tumors.

## Author summary

Primary effusion lymphoma (PEL) is a rare but aggressive malignancy, with a median survival time of 6.2 months for affected patients. Conventional treatments offer limited efficacy for PEL, and the rarity of the disease complicates the development of new drugs. Consequently, repurposing US Food and Drug Administration (FDA) approved medications for PEL treatment has become an attractive strategy. Disulfiram (DSF), a drug historically used for treating alcoholism, has demonstrated excellent pharmacokinetics, safety, and tolerance in clinical settings. In this study, DSF was found to selectively inhibit the proliferation and survival of KSHV-positive B lymphoma cells by inducing an antiviral immune response and therefore preventing the transition of KSHV from latency to the lytic stage. Deletion of TANK binding kinase 1 (TBK1) or interferon regulatory factor 3 (IRF3) disrupted innate immunity, thereby reversing the inhibitory effects of DSF on PEL cell survival and KSHV reactivation both *in vitro* and *in vivo.* Given its cost-effectiveness and FDA approval, DSF represents a promising candidate for repurposing in PEL therapy and warrants further evaluation in advanced clinical trials.

## Introduction

Kaposi’s sarcoma-associated herpesvirus (KSHV), also known as human herpesvirus 8 (HHV-8), is an oncogenic γ-herpesvirus with a large double-stranded DNA genome [1,2]. Like other herpesviruses, KSHV’s life cycle consists of two distinct phases: latent and lytic phases, both of which play crucial roles in its pathogenesis [3]. Under external stimuli, such as hypoxia, oxidative stress or inflammation, KSHV can transition from latency to lytic phase, a process known as lytic reactivation [4–7]. During reactivation, KSHV expresses viral lytic genes in a cascade, leading to the production and release of numerous virions essential for viral spread and tumor development [8]. Therefore, targeting KSHV latency represents a promising therapeutic strategy for KSHV-associated malignancies.

KSHV was first identified in 1994 within Kaposi’s sarcoma (KS) lesions in AIDS patients and has since been associated with primary effusion lymphoma (PEL), multicentric Castleman’s disease (MCD), and KSHV-associated inflammatory cytokine syndrome (KICS) [1,9–11]. Among these, PEL is highly aggressive and resistant to chemotherapy drugs that are effective against other B-cell lymphomas and therefore carries a poor prognosis [12]. The overall median and 1-year survival rates in PEL patients treated with chemotherapy were 6.2 months and 39.3%, respectively [13]. PEL primarily affects HIV-infected patients, accounting for approximately 2% to 4% lymphomas in AIDS patients [12]. Rare cases of PEL also occur in HIV-negative individuals, particularly those who are immunocompromised, such as after organ transplantation or in elderly men in regions with high KSHV prevalence, including the Mediterranean and Eastern Europe [14]. Due to its rarity, there are no large prospective clinical trials for treating PEL, making the development of effective therapies challenging. Therefore, repurposing existing FDA-approved drugs offers a faster and more cost-effective approach for PEL treatment.

Disulfiram (DSF) has minimal side effects, as demonstrated by its use as an anti-alcoholism drug for over 60 years with few adverse reactions [15]. It also exhibits favorable pharmacokinetics and tolerability [16]. Since DSF’s patent has expired, production costs have significantly decreased. In the body, DSF typically forms a complex with copper to exert cytotoxic and anticancer effects [17]. The anti-tumor properties of DSF have been shown in various cancers, including liver, pancreatic, ovarian cancers, and leukemia [18]. Several downstream effectors of DSF have been revealed. For instance, DSF inhibits proteasome activity by directly targeting NPL4 and therefore impedes breast cancer development [19]. By directly binding to and inhibiting FROUNT, a chemokine signal regulator, DSF suppresses macrophage responses and lung cancer progression [20]. Furthermore, DSF exhibits immunomodulatory effects by suppressing inflammatory signaling pathways through multiple mechanisms. It inhibits inflammation by covalently binding with a cysteine residue of MD-2, preventing STING phosphorylation, reducing NLRP3 palmitoylation, and covalently modifying or blocking the pore formation of gasdermin D (GSDMD) [21–24]. DSF also has antiviral properties either by directly targeting viral proteins or modulating the signaling pathways of host cells [25–29]. Given these characteristics, DSF might be a promising candidate for the treatment of virus-associated cancers like PEL.

In this study, we investigated the anti-tumor effects of DSF on KSHV latently infected PEL cells. We assessed DSF’s impacts on KSHV life cycle and the survival of KSHV-positive B lymphoma cells *in vitro* and *in vivo*. Our findings demonstrated that DSF effectively suppressed PEL cell proliferation and survival by inducing apoptosis. Notably, DSF inhibited the initiation and progression of PEL tumors in a xenograft mouse model. Mechanistically, DSF significantly reduced KSHV lytic reactivation in both PEL and endothelial cells, as evidenced by decreased expression of viral lytic genes and reduced virion progeny production. Furthermore, DSF inhibited KSHV reactivation by activating TANK binding kinase 1 (TBK1) and triggering antiviral immune responses. Inhibition of TBK1 or interferon regulatory factor 3 (IRF3), notably reversed DSF’s effects on KSHV reactivation, as well as PEL cell survival and progression. Overall, our study identified DSF as a novel therapeutic agent for treating PEL and preventing viral spread caused by KSHV reactivation.

## Results

### DSF inhibits PEL cell proliferation, survival and progression

**Fig 1:**
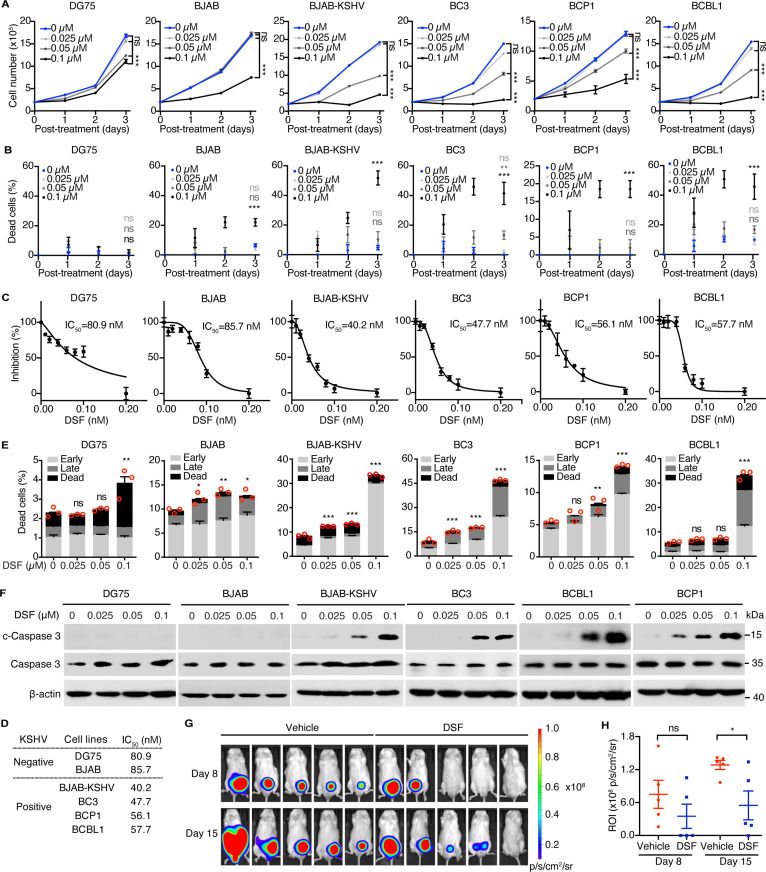
DSF inhibits PEL cell proliferation, survival and progression. (A) Proliferation curves of DG75, BJAB, BJAB-KSHV and PEL (BC3, BCBL1 and BCP1) cells treated with 0 (DMSO), 0.025, 0.05 and 0.1 μM DSF for one, two and three days. (B) Percentage of dead cells in DG75, BJAB, BJAB-KSHV and PEL cells treated with 0 (DMSO), 0.025, 0.05 and 0.1 μM DSF for one, two and three days. (C-D) Cell viability after 72 h of DSF treatment (C), with the concentration causing 50% cell death (IC_50_) induced by DSF was calculated (D). (E) Apoptosis was assessed by flow cytometry with Annexin V and PI staining of DG75, BJAB, BJAB-KSHV, and PEL cells treated with 0 (DMSO), 0.025, 0.05, and 0.1 μM DSF for 72 h. (F) Western blotting analysis in DG75, BJAB, BJAB-KSHV and PEL cells treated with 0 (DMSO), 0.025, 0.05, 0.1 μM DSF for 72 h. (G) The live imaging of PEL tumors in mice treated with vehicle or DSF after inoculation of BCBL1-Luc cells for 8 and 15 days. (H) Quantification of luminescence signals from PEL tumors in panel G, expressed as ROI (p/s/(microwatt/cm2)). *, p<0.05, **, p<0.01, ***, p<0.001, ns, not significant compared to cells treated with 0 (DMSO) μM DSF.

We first assessed the effects of DSF on multiple PEL cell lines, including BCBL1, BC3, and BCP1 cells. To provide a comparative reference, KSHV-negative Burkitt’s lymphoma cell lines BJAB and DG75 were included, as no ideal control for PEL cells exists. DSF effectively inhibited the proliferation of KSHV-positive B lymphoma cells, including BJAB cells latently infected with KSHV (BJAB-KSHV) and three PEL cell lines, demonstrating greater sensitivity than BJAB and DG75 cells (Fig 1A). Additionally, DSF treatment resulted in a significantly higher number of dead cells in BJAB-KSHV and PEL cells compared to DG75 and BJAB cells (Fig 1B). The 50% inhibitory concentration (IC_50_) of DSF for BJAB-KSHV and PEL cells ranged from 0.04 μM to 0.06 μM, while for BJAB and DG75 cells, it was 0.09 μM and 0.08 μM, respectively (Fig 1C and 1D). Consistently, DSF induced apoptosis in BJAB-KSHV and PEL cells in a dose-dependent manner, as evidenced by increased levels of cleaved Caspase 3 (c-Caspase 3) (Fig 1E and 1F). In contrast, KSHV-negative BJAB and DG75 cells exhibited greater resistance to DSF-induced apoptosis (Fig 1E and 1F). Given that DSF has been reported to modulate pyroptosis and ferroptosis, we further investigated whether either of these processes was activated in our system following DSF treatment [24,30]. A reduction in GPX4 is a hallmark of ferroptosis, while cleavage of GSDMD indicates the induction of pyroptosis [31,32]. Following DSF treatment, the expression of cleaved GSDMD remained unchanged, whereas GPX4 expression was intriguingly upregulated in BC3 and BCP1 cells (S1 Fig). These findings suggest that DSF specifically induces apoptosis, rather than ferroptosis and pyroptosis, in KSHV-positive lymphoma cells.

To further evaluate the effects of DSF on PEL survival *in vivo*, we established a xenograft mouse model by inoculating NOD-SCID mice with BCBL1 cells that stably expressed firefly luciferase (BCBL1-Luc). Treatment with DSF or vehicle commenced on day 1 post-engraftment via intraperitoneal injection and continued daily. Of note, five out of five mice (100%) in the vehicle control group developed PEL at day 8 post-inoculation, whereas only two out of five mice (40%) in the DSF-treated group developed PEL (Fig 1G). Additionally, mice treated with DSF exhibited significantly lower bioluminescence intensity than the control group on day 15 post-inoculation (Fig 1G and 1H), indicating that DSF effectively inhibited both the initiation and progression of PEL *in vivo*.

Collectively, these data demonstrated that DSF preferentially inhibited the proliferation and survival of PEL cells by inducing apoptosis both *in vitro* and *in vivo*.

### DSF inhibits KSHV reactivation in PEL cells

**Fig 2:**
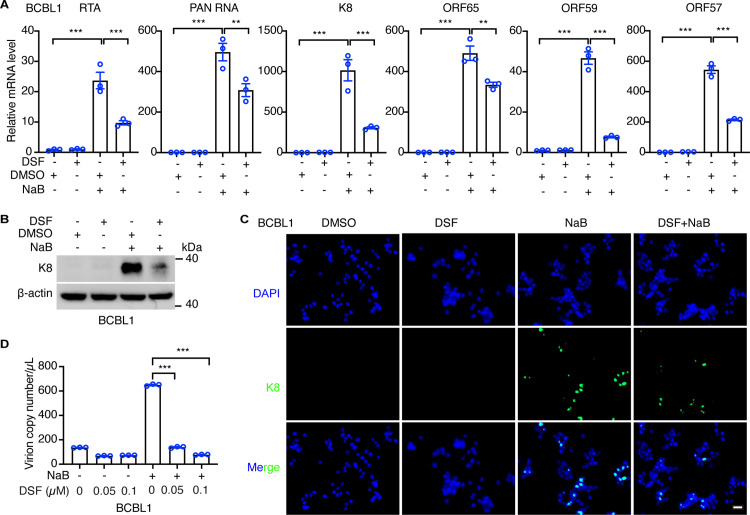
DSF inhibits KSHV reactivation in PEL cells. (A) RT-qPCR analysis of the mRNA levels of KSHV lytic genes including RTA, PAN RNA, K8, ORF65, ORF59, and ORF57 in BCBL1 cells treated with DMSO, 0.1 μM DSF, 0.5 mM sodium butyrate (NaB) or both for 72 h.(B-C) The protein level of K8 in BCBL1 cells treated with DMSO, 0.1 μM DSF, 0.5 mM sodium butyrate (NaB) or both for 72 h was examined by western blots (B) and immunofluorescence (C), respectively. Scale bar, 20 μm. (D)The produced KSHV virions in the supernatants of BCBL1 cells treated with DMSO, 0.05, 0.1 μM DSF, 0.5 mM sodium butyrate (NaB) or both for 96 h were quantified by qPCR. *, p<0.05, **, p<0.01, ***, p<0.001, ns, not significant.

DSF is shown to inhibit various viruses, such as MERS, SARS-CoV, and hepatitis C virus (HCV) [26,28,29]. Moreover, PEL cell survival is intimately related to KSHV life cycle, we thus examined the effect of DSF on KSHV reactivation in PEL cells. DSF alone barely affected the expression of KSHV lytic genes, indicating that DSF was insufficient to disrupt KSHV latency in most cells (S2A-S2D Fig). Sodium butyrate (NaB), a well-established inducer of KSHV lytic reactivation, promotes the expression of KSHV-encoded lytic proteins, such as RTA, a key regulator of reactivation, by globally inhibiting cellular deacetylases. While NaB induced a 20- to 1000-fold increase in the expression of KSHV lytic genes, including RTA, PAN RNA, K8, ORF65, ORF59 and ORF57, in BCBL1, BC3, and BCP1 cells, DSF significantly suppressed this induction (Figs 2A, S3A and S3B). Additionally, NaB markedly increased K8 protein expression, an effect that was substantially reversed by DSF treatment in all three PEL cell lines (Figs 2B and S3C). Immunofluorescence analysis further confirmed that DSF reduced NaB-induced K8 protein expression in BCBL1 cells (Fig 2C). In accordance with these results, DSF also decreased the production of KSHV progeny virions induced by NaB in the supernatants of BCBL1, BC3, and BCP1 cells (Figs 2D and S3D). Altogether, these results indicated that DSF suppresses NaB-induced lytic reactivation of KSHV in PEL cells.

### DSF inhibits KSHV reactivation in iSLK-BAC16-RGB cells

**Fig 3:**
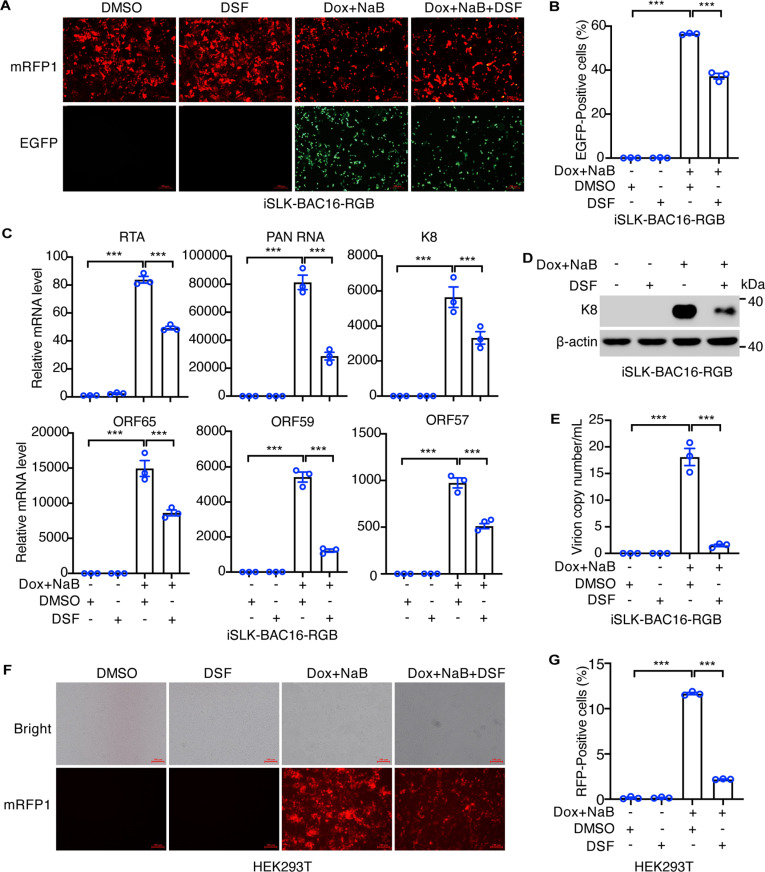
DSF inhibits KSHV reactivation in iSLK-BAC16-RGB cells. (A) iSLK-BAC16-RGB cells were treated with DMSO, 1 μM DSF, 1 μg/mL doxycycline (Dox) plus 1 mM NaB, or both for 48 h and the GFP and RFP images were captured by fluorescent microscope. (B) Flow cytometry analysis of GFP-positive iSLK- BAC16-RGB cells after the treatment of DMSO, 1 μM DSF, 1 μg/mL doxycycline (Dox) plus 1 mM NaB, or both for 48 h. (C) RT-qPCR analysis of the mRNA levels of KSHV RTA, PAN RNA, K8, ORF65, ORF59 and ORF57 in iSLK-RGB-BAC16 cells treated with 1 μM DSF, 1 μg/mL Dox plus 1 mM NaB, or both for 72 h. (D) The protein level of K8 in iSLK-RGB-BAC16 cells was examined by western blots following the treatment of DMSO, 1 μM DSF, 1 μg/mL Dox plus 1 mM NaB, or both for 72 h. (E) The produced KSHV virions in the supernatants of iSLK-RGB-BAC16 treated with DMSO, 1 μM DSF, 1 μg/mL Dox plus 1 mM NaB, or both for 96 h were detected by qPCR. (F-G) The produced KSHV virions from the supernatants of iSLK-RGB-BAC16 cells treated with DMSO, 1 μM DSF, 1 μg/mL Dox plus 1 mM NaB, or both for 96 h were used to infect HEK293T cells. The infected cells were analyzed by fluorescence microscopy (F), and flow cytometry (G) to detect RFP-positive cells after 48 h infection. *, p<0.05, **, p<0.01, ***, p<0.001, ns, not significant.

To further investigate the inhibitory effects of DSF on KSHV lytic replication, we utilized iSLK- BAC16-RGB cells. iSLK is a derivative of SLK cells that expresses a doxycycline-inducible RTA encoded by KSHV. RTA serves as the master regulator of KSHV lytic reactivation by promoting the cascade expression of viral lytic genes. BAC16-RGB is a recombinant KSHV strain that expresses a monomeric red fluorescent protein 1 (mRFP1) under the control of elongation factor 1α (EF1α) promoter, which is constitutively active in live cells. Additionally, it expresses enhanced green fluorescent protein (EGFP) under the control of the KSHV lytic PAN RNA promoter. This dual fluorescence system enables the tracking of KSHV latent and lytic phases. Fluorescent microscopy revealed that all iSLK- BAC16-RGB cells expressed mRFP1, while only few cells expressed EGFP without induction, suggesting that KSHV remained in a tightly latent phase within this system (Fig 3A). Treatment with DSF did not significantly change the expression of mRFP1 and EGFP (Fig 3A). However, simultaneous treatment with NaB and doxycycline (Dox) induced EGFP expression, which was significantly reduced by DSF treatment (Fig 3A). Flow cytometry analysis further confirmed that DSF decreased the proportion of EGFP-positive cells from approximately 56.5% to 37.2% (Fig 3B). Under uninduced conditions, fewer than 0.09% of the cells expressed EGFP, regardless of DSF treatment (Fig 3B).

We then investigated whether DSF could regulate the transcriptional program of KSHV lytic genes. Under uninduced conditions, DSF treatment slightly enhanced the transcript levels of certain KSHV lytic genes, including RTA, ORF59 and ORF57, while decreasing the expression of K8, ORF65 and PAN RNA transcripts (Fig 3C). However, these effects were marginal, and consistent with observations of EGFP expression and KSHV K8 protein levels (Fig 3C and 3D). Dual treatment with NaB and Dox significantly increased KSHV lytic gene transcription by up to 80000-fold, an effect notably reduced by DSF (Fig 3C). Additionally, the induction of KSHV K8 protein by NaB and Dox was consistently diminished by DSF (Fig 3C and 3D).

To further determine whether DSF reduced the production of infectious KSHV virions, we first performed qPCR to quantify the copies of viral genome released into the culture supernatants. While viral genomes were barely detected under normal conditions, regardless of DSF treatment, the production of KSHV virions induced by NaB and Dox was significantly decreased following DSF treatment (Fig 3E). Additionally, culture supernatants from iSLK-RGB-BAC16 cells, collected on day 4 post-induction with NaB and Dox, were used to infect HEK293T cells, which are highly susceptible to KSHV infection. Cells expressing mRFP1 were examined to determine the relative titer of KSHV infectious virions (Fig 3F). DSF treatment reduced the production of infectious virions by 5.3-fold (Fig 3G).

Taken together, these results indicated that DSF potently suppressed KSHV lytic reactivation in both PEL and endothelial cells.

### DSF triggers innate-immune responses

**Fig 4:**
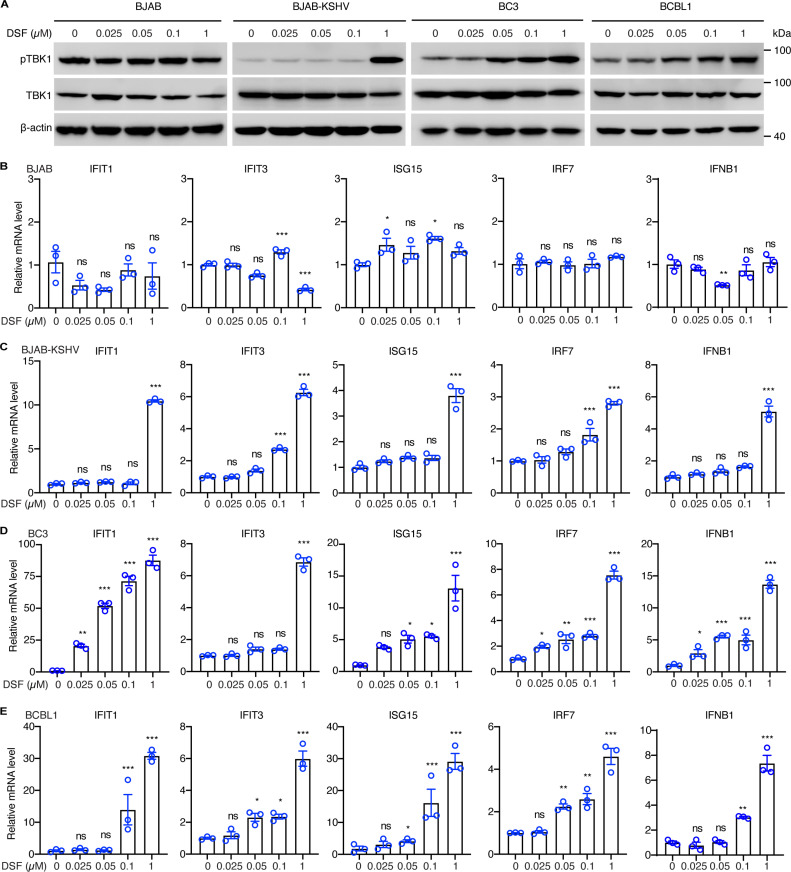
DSF triggers innate immune responses. (A) Western blotting analysis of BJAB, BJAB-KSHV, BC3 and BCBL1 cells treated with 0 (DMSO), 0.025, 0.05, 0.1, 1 μM DSF for 24 h. (B-E) RT-qPCR analysis of the mRNA levels of IFIT1, IFIT3, ISG15, IRF7 and IFNB1 in BJAB (B), BJAB-KSHV (C), BC3 (D) and BCBL1 (E) cells treated with 0 (DMSO), 0.025, 0.05, 0.1, 1 μM DSF for 24 h. *, p<0.05, **, p<0.01, ***, p<0.001, ns, not significant compared to 0 (DMSO) μM DSF.

Previous studies have demonstrated that DSF can efficiently modulate the immune system in cancer cells [20,22,33]. Given that DSF effectively impeded KSHV reactivation in both PEL and endothelial cells, we speculated that DSF might activate antiviral immune responses. TBK1, a central node protein in antiviral innate immunity, was evaluated for its activity following DSF treatment. Specifically, we assessed the phosphorylation of TBK1 at serine 172 (pTBK1-S172), a marker of TBK1 activation. DSF significantly activated TBK1 in PEL cells including BC3 and BCBL1 in a dose-dependent fashion (Fig 4A). Notably, DSF at a concentration of 0.05 μM was sufficient to induce TBK1 activation in BC3 and BCBL1 cells (Fig 4A). In contrast, DSF activated TBK1 in BJAB-KSHV cells only at 1 μM, indicating that BJAB-KSHV cells were less responsive compared to KSHV-positive PEL cells (Fig 4A). Moreover, TBK1 in BJAB cells was minimally affected by DSF across concentrations ranging from 0 to 1 μM (Fig 4A). These results suggested that DSF-mediated activation of TBK1 was indirect and dependent on KSHV. To further validate these results, we examined the downstream effectors of TBK1 following DSF treatment. DSF treatment consistently upregulated the mRNA levels of IFNB1 and several interferon-stimulated genes (ISGs) including IFIT1, IFIT3, ISG15, and IRF7 in KSHV-positive BC3 and BCBL1 cells but not in BJAB cells, in a dose-dependent manner (Fig 4B, 4D and 4E). Moreover, 1 μM DSF, rather than other concentrations, significantly increased the transcript expression of IFNB1 and ISGs in BJAB-KSHV cells, consistent with TBK1 activation (Fig 4C). Collectively, these findings indicated that DSF preferentially triggered the antiviral innate immune pathway by activating TBK1 in KSHV-positive rather than KSHV-negative lymphoma cells.

Diethyldithiocarbamate (DDTC), an active metabolite of DSF, has been reported to inhibit NF-κB, a pathway crucial for PEL cell survival [34]. We further examined the effects of DSF on NF-κB activity. As expected, DSF suppressed NF-κB signaling pathway in PEL cells (S4 Fig). However, this suppression is well-characterized and less pronounced compared to DSF-mediated TBK1 activation. We thus focused on investigating DSF’s activation of TBK1.

### DSF suppresses KSHV reactivation by triggering innate-immune responses

**Fig 5:**
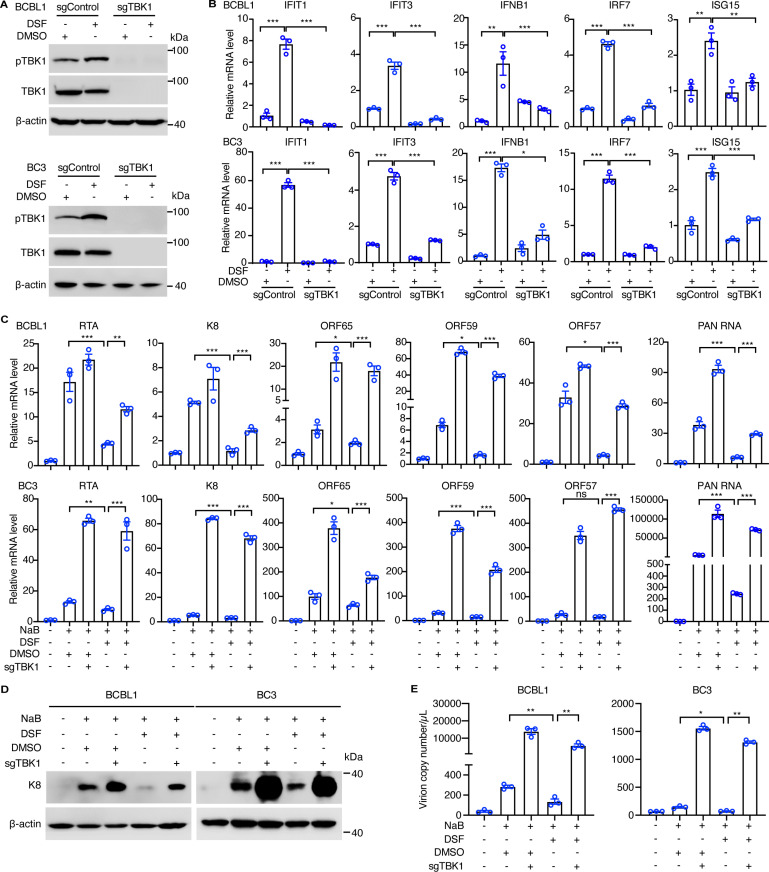
DSF suppresses KSHV reactivation by activating innate-immune responses. (A) Western blotting analysis of BCBL1 and BC3 wildtype and TBK1 knockout cells with 0 (DMSO) or 1 μM DSF treatment for 24 h. (B) RT-qPCR analysis of the mRNA levels of ISGs, including IFIT1, IFIT3, IFNB1, IRF7 and ISG15, in BCBL1 and BC3 wildtype or TBK1 knockout cells with 0 (DMSO) or 1 μM DSF treatment for 24 h. (C) RT-qPCR analysis of the mRNA levels of KSHV lytic genes, including RTA, K8, ORF65, ORF59, ORF57 and PAN RNA in BCBL1 and BC3 wildtype and TBK1 knockout cells treated with 0 (DMSO), 0.1 μM DSF, 0.5 mM NaB, or both for three days. (D) Western blotting analysis of BCBL1 and BC3 wildtype and TBK1 knockout cells treated with 0 (DMSO), 1 μM DSF, 0.5 mM NaB, or both for three days. (E) RT-qPCR detection of KSHV virions in the supernatants of BCBL1 and BC3 wildtype and TBK1 knockout cells treated with 0 (DMSO), 0.1 μM DSF, 0.5 mM NaB, or both for three days. *, p<0.05, **, p<0.01, ***, p<0.001, ns, not significant.

To investigate whether DSF inhibits KSHV reactivation and PEL cell survival by activating TBK1, we suppressed TBK1 by either genomic disruption or pharmacological inhibition. As expected, both TBK1 knockout or treatment with GSK8612, a potent TBK1 inhibitor, efficiently blocked DSF-induced TBK1 activation in BCBL1 and BC3 cells (Figs 5A and S5A). Consistently, DSF treatment significantly increased the mRNA levels of IFNB1 and ISGs, including IFIT1, IFIT3, ISG15, and IRF7, in BCBL1 and BC3 cells (Figs 5B and S5B). This increase was markedly attenuated by TBK1 knockout or GSK8612 treatment (Figs 5B and S5B). Additionally, TBK1 deletion significantly enhanced NaB-induced KSHV reactivation, as evidenced by the increased expression of KSHV lytic genes and augmented production of virions (Fig 5C-5E). These results highlighted the crucial role of TBK1 in preventing KSHV lytic replication from latency. Consistently, TBK1 knockout or treatment with GSK8612 significantly reversed DSF’s inhibition of NaB-induced KSHV lytic gene transcription, including RTA, PAN RNA, K8, ORF65, ORF59, and ORF57, in BCBL1 and BC3 cells (Figs 5C and S5C). Furthermore, TBK1 knockout or GSK8612 treatment reversed DSF’s inhibition of NaB-induced K8 protein expression (Figs 5D and S5D). Moreover, TBK1 knockout or GSK8612 treatment also reduced the production of NaB-induced KSHV virions following DSF treatment in BCBL1 and BC3 cells (Figs 5E and S5E). Collectively, these findings indicated that DSF inhibited KSHV lytic reactivation in PEL cells by activating innate immune responses.

### DSF inhibits PEL proliferation, survival and progression by activating TBK1

**Fig 6:**
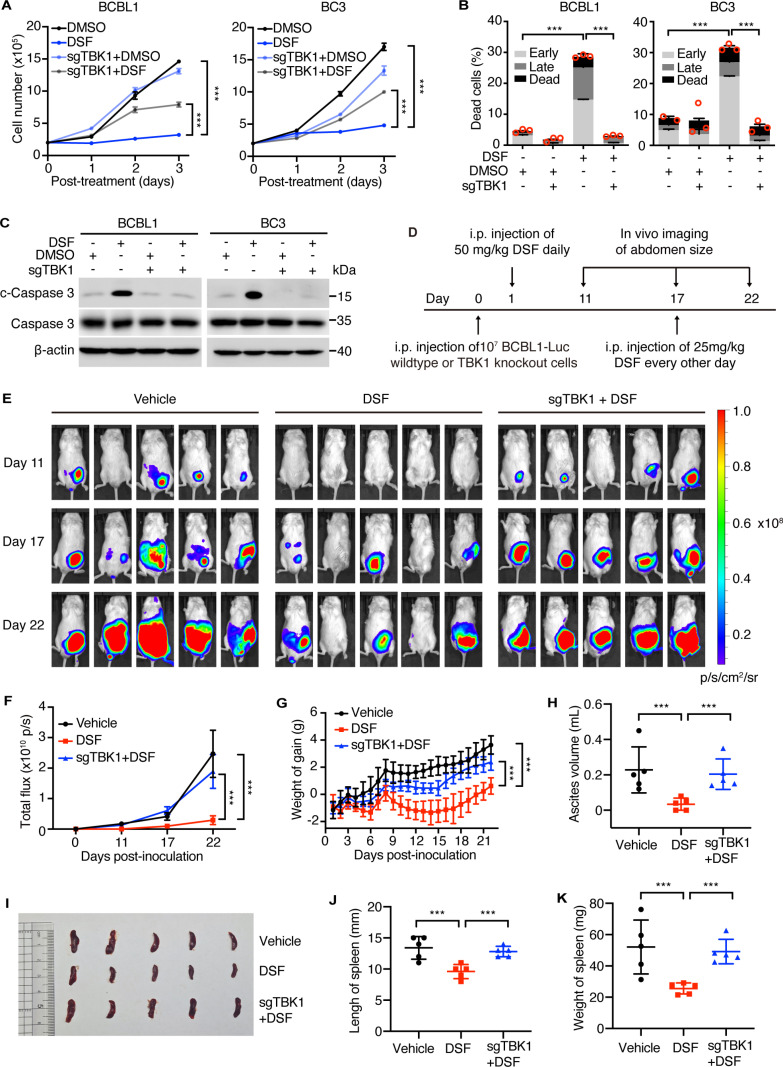
DSF suppresses PEL proliferation, survival and progression by activating TBK1. (A) Proliferation curves of BCBL1 and BC3 wildtype and TBK1 knockout cells with 0 (DMSO) or 0.1 μM DSF for three days. (B) Apoptotic cells were detected by flow cytometry with Annexin V and PI staining in BCBL1 and BC3 wildtype and TBK1 knockout cells following 0 (DMSO) or 0.1 μM DSF treatment for three days. (C) Western blotting analysis of BCBL1 or BC3 wildtype and TBK1 knockout cells with 0 (DMSO) or 0.1 μM DSF treatment for three days. (D) Schematic illustration of the animal experiment. (E) Live imaging of mice engrafted with BCBL1-Luc wildtype or TBK1 knockout cells that were treated with vehicle or indicated concentrations of DSF for 11, 17, and 22 days. (F) Quantification of luminescence signals from PEL tumors in panel (E). (G) Volume of ascites extracted from mice at day 22 post-inoculation. (H) Daily weight gain of mice. (I-K) Photographs (I), length (J), and weights (K) of spleen of mice at day 22 post-inoculation.

Next, we explored whether DSF inhibited PEL proliferation and survival through activation of innate immune responses. As expected, TBK1 suppression by knockout or GSK8612 treatment impaired DSF’s inhibitory effects on the proliferation of BCBL1 and BC3 cells (Figs 6A and S6A). Additionally, TBK1 knockout or GSK8612 treatment notably reduced the percentage of apoptotic cells and the levels of c-Caspase 3 protein induced by DSF in BCBL1 and BC3 cells (Figs 6B, 6C, S6B and S6C). These results strongly indicated that DSF inhibited PEL proliferation and survival by mediating TBK1 activation.

We then assessed the *in vivo* dependence of DSF on TBK1 in inhibiting PEL cell survival in a mouse model. To this end, we constructed a BCBL1-Luc cell line with stable TBK1 knockout (BCBL1-Luc-sgTBK1). We verified that the bioluminescence signals of BCBL1-Luc wildtype cells were comparable to BCBL1-Luc-sgTBK1 cells, allowing us to evaluate the dependence of DSF on TBK1 in regulating PEL growth *in vivo* (S6D Fig). Both wild-type and TBK1 knockout BCBL1-Luc cells were engrafted into NOD-SCID mice and either vehicle or DSF treatment was initiated the following day (Fig 6D). Consistently, all five mice (100%) in the vehicle control group developed PEL by day 22 post-inoculation, compared to three out of five mice (60%) in the DSF-treated group (Fig 6E). In contrast, all five mice (100%) with BCBL1-Luc-sgTBK1 cells developed PEL by day 22, even with DSF treatment (Fig 6E). Additionally, DSF treatment resulted in significantly lower bioluminescence signal intensity compared to the control group, an effect that was reversed by TBK1 knockout (Figs 6E, 6F and S6E). PEL development typically causes abnormal weight gain, partially due to ascites formation. DSF treatment reduced the weight gain observed in the control group, an effect that was reversed by TBK1 knockout (Figs 6G and S6F). Furthermore, DSF treatment markedly reduced ascites volume and spleen enlargement caused by PEL, with these effects abolished by TBK1 deletion (Fig 6H-6K). Solid tumor metastases to the peritoneum and retroperitoneum were smaller following DSF treatment, but this effect was reversed by TBK1 knockout (S6G Fig). Neither TBK1 knockout nor DSF treatment caused significant changes in organs such as the heart, liver, spleen, lung, or kidney, indicating the safety of DSF at these concentrations (S6H Fig). Taken together, these results demonstrated that TBK1 mediated DSF’s inhibition of PEL survival both *in vivo* and *in vitro*. TBK1 deletion abolished DSF’s suppressive effects on PEL development.

### IRF3 mediates DSF suppression of KSHV reactivation and PEL cell survival

**Fig 7:**
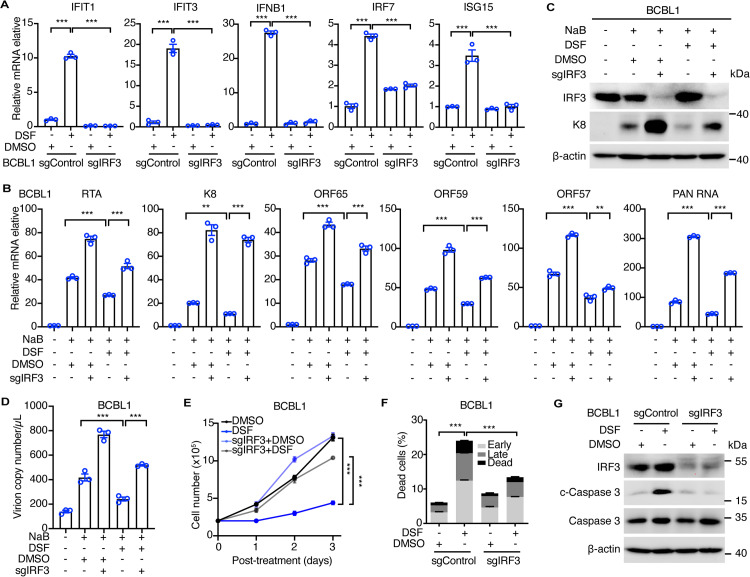
IRF3 mediates DSF suppression of KSHV reactivation and PEL cell survival. (A) RT-qPCR analysis of the mRNA levels of ISGs, including IFIT1, IFIT3, IFNB1, IRF7, and ISG15 in BCBL1 wildtype or IRF3 knockout cells with 0 (DMSO) or 1 μM DSF treatment for 24 h. (B) RT-qPCR analysis of the mRNA levels of KSHV lytic genes, including RTA, K8, ORF65, ORF59, ORF57 and PAN RNA in BCBL1 wildtype and IRF3 deletion cells treated with 0 (DMSO), 0.1 μM DSF, 0.5 mM NaB, or both for three days. (C) Western blotting analysis of BCBL1 wildtype and IRF3 knockout cells treated with 0 (DMSO), 1 μM DSF, 0.5 mM NaB, or both for three days. (D) RT-qPCR detection of KSHV virions in the supernatants of BCBL1 wildtype and IRF3 knockout cells treated with 0 (DMSO), 0.1 μM DSF, 0.5 mM NaB, or both for three days. (E) Proliferation curves of BCBL1 wildtype and IRF3 knockout cells with 0 (DMSO) or 0.1 μM DSF for three days. (F) Apoptotic cells were detected by flow cytometry with Annexin V and PI staining in BCBL1 wildtype and IRF3 knockout cells following 0 (DMSO) or 0.1 μM DSF treatment for three days. (G) Western blotting analysis of BCBL1 wildtype and IRF3 knockout cells with 0 (DMSO) or 1 μM DSF treatment for 24 h. *, p<0.05, **, p<0.01, ***, p<0.001, ns, not significant.

IRF3 is the downstream effector of TBK1 and plays a crucial role in the immune defense against viral infection. To further corroborate our findings, we constructed BCBL1 cell lines with IRF3 knockout. Consistent with observations in TBK1 knockout cells, deletion of IRF3 led to a reduction in the expression of IFNB1 and ISG genes induced by DSF treatment, including IFIT1, IFIT3, IRF7, and ISG15, in BCBL1 cells (Fig 7A). Moreover, IRF3 knockout significantly reversed DSF’s inhibition of NaB-induced KSHV lytic gene transcription, including RTA, PAN RNA, K8, ORF65, ORF59, and ORF57 (Fig 7B). IRF3 knockout also reversed DSF’s inhibition of NaB-induced K8 protein expression and reduced the production of NaB-induced KSHV virion progeny following DSF treatment (Fig 7C and 7D). Notably, IRF3 depletion attenuated DSF’s inhibitory effects on BCBL1 cell proliferation (Fig 7E). Additionally, IRF3 knockout significantly reduced the proportion of apoptotic cells and the levels of c-Caspase 3 protein induced by DSF in BCBL1 cells (Fig 7F and 7G). Taken together, these results suggested that DSF inhibited KSHV reactivation and promoted PEL cell apoptosis through interferon-mediated responses.

## Discussion

Despite advances in our understanding of cancer biology, malignant diseases continue to impose a significant global burden. PEL is a unique type of tumor linked to KSHV latent infection, frequently observed in individuals with compromised immune systems, such as those with HIV [2,12]. The 1-year survival rate for PEL patients is 39.3%, and no specific and effective treatments are currently available [13,35]. The high mortality rate of PEL highlights the urgent need for innovative therapeutic treatments. Given the high costs, lengthy development periods, and high failure rates associated with new drug discovery, repurposing existing medications approved for various diseases as potential anti-cancer therapies offers a more cost-effective and expedited alternative [36,37]. This approach leverages established clinical formulations and documented patient tolerability.

DSF is an attractive candidate in cancer therapy, as it has been used for treating alcoholism for over six decades and is shown to be effective in killing diverse types of cancer in preclinical studies through multiple mechanisms [16,38]. Studies have demonstrated that DSF potently inactivates proteasomes, inhibits the NF-κB signaling pathway, activates pro-apoptotic JNK and MAPK pathways, induces endoplasmic reticulum stress, promotes oxidative stress, and enhances the efficiency of immunotherapy, and so on [19,39–44]. Here, we found that DSF potently inhibits PEL cell proliferation and survival by specifically inducing apoptosis. Notably, DSF suppresses the initiation and progression of PEL tumors by activating TBK1. Intriguingly, DSF activation of TBK1 is exclusively observed in KSHV infected cells, but not in KSHV-negative lymphoma cells, suggesting that TBK1 activation by DSF is indirect and dependent on KSHV infection. One possible explanation is that DSF directly binds to and targets viral proteins that negatively regulate TBK1 activity, thereby enabling selective TBK1 activation in KSHV-positive cells. Although DSF also impairs the proliferation of KSHV-negative lymphoma cells, this effect is less pronounced. Since DSF does not activate TBK1 in KSHV-negative lymphoma cells at the concentrations tested, an alternative mechanism independent of TBK1 likely accounts for DSF’s inhibition of KSHV-negative lymphoma cell proliferation. Consistent with our observations, previous studies showed that DSF could inhibit the progression of hematocarcinoma including leukemia and lymphoma [45,46]. Moreover, DDTC, an active metabolite of DSF, significantly suppresses PEL growth by inhibiting NF-κB and inducing apoptosis [34]. Indeed, we observed that DSF treatment also inactivates NF-κB signaling pathway, albeit it is less effective than activating TBK1. Whether the induction of PEL cell death by DSF is mediated by NF-κB awaits further investigation.

In PEL tumor cells, KSHV typically remains in a latent phase with minimal viral gene expression, which allows the virus to evade host immune surveillance, presenting a significant challenge for treating KSHV-associated tumors [6]. Several studies, including our findings, suggest that the induction of KSHV lytic reactivation from latency could be a promising therapeutic strategy [6,47–49]. However, this approach risks releasing and spreading viral particles throughout the body. Thus, an ideal therapeutic drug should not only be cytotoxic but also inhibit KSHV reactivation. Indeed, several studies have shown that inhibiting KSHV reactivation induces apoptosis, leading to the death of PEL cells [50,51]. Our study found that DSF prevents KSHV reactivation triggered by external stimuli through activating TBK1, which subsequently initiates antiviral innate immunity and enhances immune surveillance. Inhibition of TBK1 or its downstream effector IRF3 abolisheds DSF’s inhibitory effect on KSHV reactivation and PEL cell proliferation and survival. DSF not only inhibits the survival of KSHV-positive B lymphoma cells but also reduces the production and spread of infectious virion progeny, indicating DSF as a potentially ideal therapeutic agent for PEL. Since most PEL cells are latently infected by KSHV and express minimal viral lytic genes, it is important to note that the inhibition of KSHV lytic reactivation from latency by DSF only marginally contributes to the decreased survival of PEL cells under normal conditions. However, factors in the cancer microenvironment, such as hypoxia, oxidative stress, and inflammation, can trigger KSHV reactivation [4,5,7]. Therefore, it is possible that DSF may suppress PEL development and progression in these environments, or *in vivo*. The antiviral activities of DSF are also observed in other viruses. It is shown that DSF reactivates latent HIV-1 and subsequently targets viral reservoirs by activating the AKT signaling pathway [52]. Additionally, DSF alters the conformation of hepatitis C virus (HCV) non-structural protein 5A (NS5A) by binding to its zinc finger site and therefore inhibits HCV assembly and replication [28]. Furthermore, DSF inhibits severe acute respiratory syndrome coronavirus (SARS-CoV) assembly and replication by targeting and inhibiting the papain-like protease [29].

Altogether, we explored the possibility of DSF as a remedy for virus-associated diseases by utilizing KSHV latently infected PEL cells as a model. Our findings showed that DSF robustly induces the cytotoxicity in PEL cells and inhibits KSHV reactivation in both PEL and endothelial cells, which is highly dependent on the activation of TBK1 and antiviral innate immunity. All these establish DSF as a potential therapeutic agent for treating PEL.

## Materials and methods

### Ethics statement

All animal-related experiments adhered to the guidelines established by the Animal Use and Care Management Advisory Committee of Hunan Normal University (2024451). NOD/SCID mice, aged around 5 weeks, were housed under pathogen-free conditions.

### Cell lines and cell culture

DG75, BJAB, BJAB-KSHV, and three PEL cells (BC3, BCBL1, and BCP1) were cultured in RPMI1640 medium (Gibco) supplemented with 10% fetal bovine serum (ExCell) and antibiotics (100 μg/mL streptomycin and 100 μg/mL penicillin). iSLK-BAC16-RGB cells were maintained in Dulbecco’s Modified Eagle’s Medium (DMEM) (Gibco) supplemented with 10% fetal bovine serum (ExCell), 1% penicillin-streptomycin, 600 μg/mL hygromycin, 1 μg/mL puromycin, and 250 μg/mL G418. HEK293T cells were cultured in DMEM (Gibco) supplemented with 10% FBS (ExCell), 100 μg/mL streptomycin and 100 μg/mL penicillin. All cells were cultured in a humidified incubator at 37°C with 5% CO_2_.

### Cell proliferation and cell viability assay

Cells were seeded at a density of 2x10^5^ per well in 12-well plates. Viable and non-viable cells were assessed daily. Specifically, cells were stained with a 0.4% trypan blue solution (Yesen, 40207ES20) in a 1:1 ratio. Viable cells remained unstained, while dead cells appeared blue and were counted using a hemocytometer. The total cell count was calculated by applying the dilution factor.

### Apoptosis assay

Cells were seeded at a density of 2x10^5^ cells per well in a 12-well plate. After treatment, cells were collected by centrifugation at 1500 rpm for 5 minutes and washed once with PBS. Subsequently, cells were stained with propidium iodide (PI) and phycoerythrin-cyanine 7-conjugated Annexin V (Elabscience, E-CK-A217) at room temperature for 15 minutes in the dark. Apoptotic cells were detected using flow cytometry (Thermo Fisher, Attune NxT), with Annexin V staining indicating early apoptosis, PI staining indicating dead cells, Annexin V and PI staining indicating late apoptosis, and unstained cells considered live. Data were analyzed with FlowJo V10 software (FlowJo, LLC, Ashland, OR).

### Quantification of KSHV genome copy number

The supernatants from treated cells were collected and centrifuged at 3000 g for 3 minutes to remove cell debris. Next, 100 U/mL of DNase I (Invitrogen, P30727) was added to the supernatants and incubated at 37°C for 30 minutes to digest DNA. Following this, 10 mM EDTA was added, and the mixture was incubated at 65°C for 10 minutes. qPCR was conducted by using specific primers for KSHV ORF65 (Forward: 5’-ATATGTCGCAGGCCGAATA-3’, Reverse: 5’-CCACCCATCCTCCTCAGATA-3’). The standard curve for qPCR was generated by using a pCDH plasmid containing KSHV ORF65 coding sequence as the template, which was then used to calculate the KSHV genome copy number in the supernatants.

### Generation of knockout cell lines

Knockout was achieved using the CRISPR/Cas9 system. A single guide RNA (sgRNA) targeting TBK1, IRF3 and IRF7 was designed with the CRISPOR tool (http://crispor.tefor.net) and cloned into the pLentiCRISPR-V2 vector. The sgRNA expression vector was then packaged into lentiviruses that were used to infect PEL cells. 24 hours post-infection, selection was performed using 2 μg/mL puromycin for 4 days to eliminate uninfected cells. Surviving cells were subsequently diluted to single cells that were re-plated into 96-well plates for clonal expansion. The target sequences of sgTBK1, sgIRF3 and sgIRF7 used was listed in S1 Table.

### Immunofluorescent assay (IFA)

Treated PEL cells were collected and centrifuged at 1500 rpm for 3 minutes to pellet the cells. The pellets were washed once with PBS, fixed with 4% paraformaldehyde at room temperature for 30 minutes, and then washed again with PBST (PBS containing 0.1% Tween-20), followed by centrifugation at 1500 rpm for 3 minutes to obtain the cell pellets. Following this, the cells were centrifuged at 1500 rpm for 3 minutes to obtain the cell pellets. The cells were permeabilized with 0.25% Triton X-100 in PBST for 15 minutes at room temperature and subsequently blocked with 1% BSA in PBST for 30 minutes at 37°C. The cells were then incubated overnight at 4°C with anti-K8 monoclonal antibody (Santa Cruz, F33P1) diluted 1:300. After washing with PBST, the cells were centrifuged at 2000 rpm for 3 minutes and incubated with Alexa Fluor 488-conjugated goat anti-mouse secondary antibody (company, cat. no) at 37°C for 60 minutes in the dark. Following another wash with PBST and centrifugation at 1500 rpm for 3 minutes, DAPI staining (Solarbio, C0060) was performed at room temperature for 10 minutes. The cells were then washed with PBST, centrifuged at 2000 rpm for 3 minutes, and a small amount of PBST was used to disperse the pellets before applying it evenly to a cover glass. Finally, the cover glass was mounted on a coverslip using anti-quench mounting buffer (Solarbio, S2100), and the cells were visualized using a fluorescence microscope (Zeiss, M2).

### RNA isolation and RT-qRCR

Cells were lysed using TRIzol Reagent (TransGen Biotech, ET111-01), and total RNA was extracted with an RNA extraction kit (TransGen Biotech, ET111) following the manufacturer’s instructions. Next, 500 μg total RNA was reverse-transcribed into cDNA using a cDNA synthesis kit (TransGen Biotech, P40719). qPCR analysis of the cDNA was performed with SYBR Green dye (TransGen Biotech, AUQ-01), and all qPCR reactions were conducted in triplicate. Relative gene expression was calculate by normalizing to β-actin using the formula 2-^ΔΔCt^. The sequences of RT-qPCR primers were provided in S1 Table.

### Western blotting analysis

Cells were lysed with 1x Laemmli buffer (62.5 mM Tris-HCl, 10% glycerol, 2% SDS, 2.5% β-mercaptoethanol, and 0.01% bromophenol blue) and boiled at 95°C for 10 minutes to prepare whole cell lysates. The lysates were then separated by SDS polyacrylamide gel electrophoresis and transferred to a nitrocellulose membrane. The membrane was blocked with 3% skim milk at room temperature for 1.5 hours, incubated with primary antibodies at 4°C overnight, and subsequently incubated with the horseradish peroxidase (HRP)-conjugated secondary antibodies at room temperature for 1 hour. Signal detection was performed using West Dura ECL substrate (FTC lifetime, 047-500 mL), and images were captured using the ChemiDoc MP Imaging System (Bio-Rad). Primary antibodies used included anti-β-actin (ABclonal, AC026), anti-TBK1 (Cell Signaling Technology, 2880), anti-pTBK1 (Cell Signaling Technology, 2880), anti-cleaved Caspase 3 (Cell Signaling Technology, 9664), anti-p65 (Cell Signaling Technology, 8242), anti-p-p65 (Cell Signaling Technology, 3033), anti-IRF3 (Cell Signaling Technology, 4302), anti-GPX4 (Proteintech, 67763), anti-Caspase 3 (Abclonal, A19654), anti-GSDMD (Santa Cruz, sc-81868) and anti-K8 (Santa Cruz, F33P1).

### Animal Experiments

To assess the effects of DSF on PEL progression, NOD/SCID mice were randomly assigned to two groups. Each mouse received an intraperitoneal injection of 200 μL DMEM containing 1x10^7^ BCBL1 cells transduced with firefly luciferase (BCBL1-Luc). One day after cell injection, the experimental group was administered daily intraperitoneal injections of 50 mg/kg DSF (MCE, HY-B0240), while the control group received an equivalent volume of the corresponding solvent, which consisted of 50% PEG300 and 5% DMSO in saline. To reveal whether DSF suppresses PEL progression by activating TBK1, 1x10^7^ BCBL1-Luc wildtype or TBK1 Knockout cells were injected intraperitoneally into separate NOD/SCID mice. One day post-inoculation, mice received 50 or 25 mg/kg DSF or the corresponding solvent. To visualize PEL tumors, D-luciferin (PerkinElmer, 122799) was intraperitoneally injected at a final concentration of 50 mg/kg and allowed to incubate for 8 minutes. Subsequently, mice were imaged using the IVIS Lumina LT system for 10 seconds. Region of interest (ROI) signals and total flux were analyzed using Living Image software (IVIS Imaging System) based on the formula (p/s)/(microwatt/cm2) and p/s, respectively.

### Statistical analysis

Unless otherwise specified, data are presented as the mean ± standard error of the mean (SEM) from at least three independent experiments. Statistical analysis between two groups was performed using a two-tailed t-test, while one-way ANOVA was employed for comparisons involving more than two groups, unless stated otherwise. A p-value of ≤ 0.05 was considered statistically significant. The symbols “*”, “**”, and “***” denote p-values less than 0.05, 0.01, and 0.001, respectively, while “ns” indicates no statistically significant difference.

## Supporting information

S1 Fig DSF treatment fails to induce the pyroptosis and ferroptosis of PEL cells.
The protein level of full-length (FL) Gasdermin D (GSDMD), the cleaved N-terminal (N) GSDMD and GPX4 was examined by western blots following the treatment with different concentrations of DSF for three days in BC3, BCBL1 and BCP1 cells.
(TIF)

S2 Fig DSF has minimal effects on KSHV reactivation under normal conditions.
(A) RT-qPCR analysis of the mRNA levels of KSHV RTA, PAN RNA, K8, ORF65 and ORF59 in BJAB-KSHV, BC3, BCP1, and BCBL1 cells treated with 0, 0.025,0.05,0.1 μM DSF or 0.5 mM NaB for 72 h. *, p<0.05, **, p<0.01, ***, p<0.001, ns, not significant compared to 0 μM DSF.
(TIF)

S3 Fig DSF inhibits NaB-induced KSHV reactivation in PEL cells.
(A-B) RT-qPCR analysis of the mRNA levels of KSHV RTA, PAN RNA, K8, ORF65, ORF59 and ORF57 in BC3 (A) and BCP1 (B) cells treated with 0.1 μM DSF,0.5 mM NaB or both for 72 h. (C) The protein level of K8 was examined by western blots following the treatment of 0.1 μM DSF, 0.5 mM NaB or both for 72 h in BC3 and BCP1 cells. (D)The produced KSHV virions in the supernatants of BC3 and BCP1 cells treated with 0.1 μM DSF, 0.5 mM NaB or both for 96 h was detected by qPCR. *, p<0.05, **, p<0.01, ***, p<0.001, ns, not significant.
(TIF)

S4 Fig DSF inhibits NF-κB activation in PEL cells.
The protein level of the phosphorylation of p65 at S276 and total p65 following the treatment of 1 μM DSF for 24 h in BJAB, BJAB-KSHV, BC3 and BCBL1 cells.
(TIF)

S5 Fig DSF suppresses KSHV reactivation by activating innate immune responses.
(A) Western blotting analysis of BCBL1 and BC3 cells treated with 0 (DMSO), 1 μM DSF, 0.2 μM GSK8612 or both for 24h. (B) RT-qPCR analysis of the mRNA levels of IFNB1 and ISGs including IFIT1, IFIT3, IRF7, and ISG15 in BCBL1 and BC3 cells treated with 1 μM DSF, 0.2 μM GSK8612 or both for 24 h. (C) RT-qPCR analysis of the mRNA levels of KSHV RTA, PAN RNA, K8, ORF65, ORF59 and ORF57 in BCBL1 and BC3 cells treated with 0.5 mM NaB, 0.1 μM DSF, 0.5 μM GSK8612, both or three of them for 72 h. (D) The protein level of K8 was examined by western blots following the treatment of 0.5 mM NaB, 0.1 μM DSF, 0.5 μM GSK8612, both or three of them in BCBL1 and BC3 cells for 72 h. (E) The produced KSHV virions in the supernatants of BCBL1 and BC3 cells treated with 0.5 mM NaB, 0.1 μM DSF, 0.5 μM GSK8612, both or three of them for 96 h was quantitated by qPCR. *, p<0.05, **, p<0.01, ***, p<0.001, ns, not significant.
(TIF)

S6 Fig DSF suppresses PEL proliferation, survival and progression by activating TBK1. (A) Proliferation curves of BC3 and BCBL1cells treated with 0.1 μM DSF, 0.2 μM GSK8612 or both for continual three days. (B) Apoptosis was detected by flow cytometry with Annexin V and PI staining in BCBL1and BC3 cells treated with 0.1 μM DSF, 0.2 μM GSK8612 or both for three days. (C) Western blotting detection in BCBL1 and BC3 cells treated with 0.1 μM DSF, 0.5 μM GSK8612 or both for three days. (D) Bioluminescence signal of BCBL1-Luc wildtype and TBK1 knockout cells in a 96-well plate. (E) Quantification of luminescence signals from PEL tumors in individual mouse. (F) Body weights of mice. (G) Photograph of solid tumors in the peritoneum and retroperitoneum. (H) Photograph of organs including heart, lung, liver, kidney, pancreas, and spleen of mice engrafted with BCBL1-Luc wildtype or TBK1 knockout cells and treated with vehicle or DSF.
(TIF)

S1 Table Primer sequences. (DOCX)
