# Supplementary figures and images for: Repurposing alcohol-abuse drug disulfiram for the treatment of KSHV-infected primary effusion lymphoma by activating antiviral innate immunity
Source: PLoS Pathog. 2025 Mar 4;21(3):e1012957. doi: 10.1371/journal.ppat.1012957 (PMC11922253; doi:10.1371/journal.ppat.1012957)

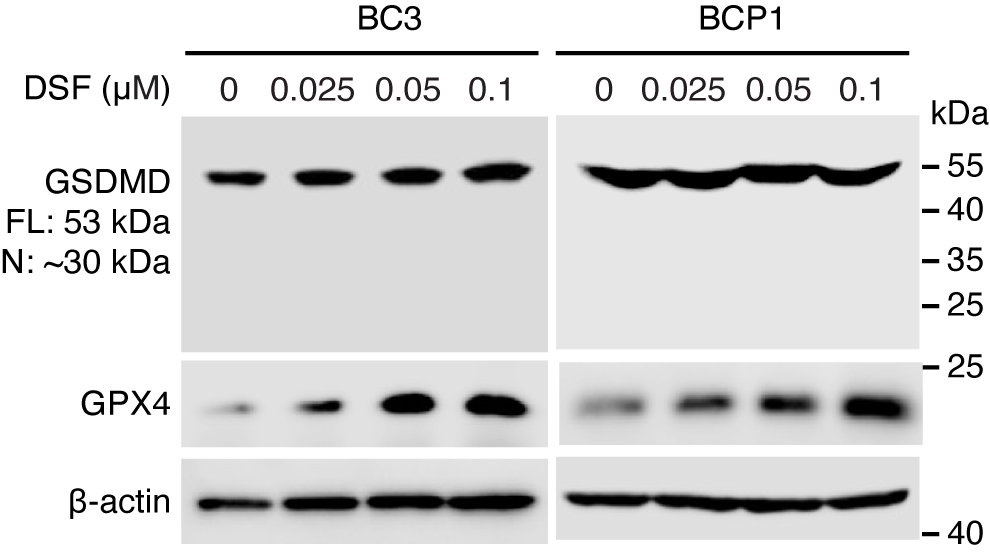

Supplement: S1 Fig — The protein level of full-length (FL) Gasdermin D (GSDMD), the cleaved N-terminal (N) GSDMD and GPX4 was examined by western blots following the treatment with different concentrations of DSF for three days in BC3, BCBL1 and BCP1 cells. (TIF) [file ppat.1012957.s001.tif]

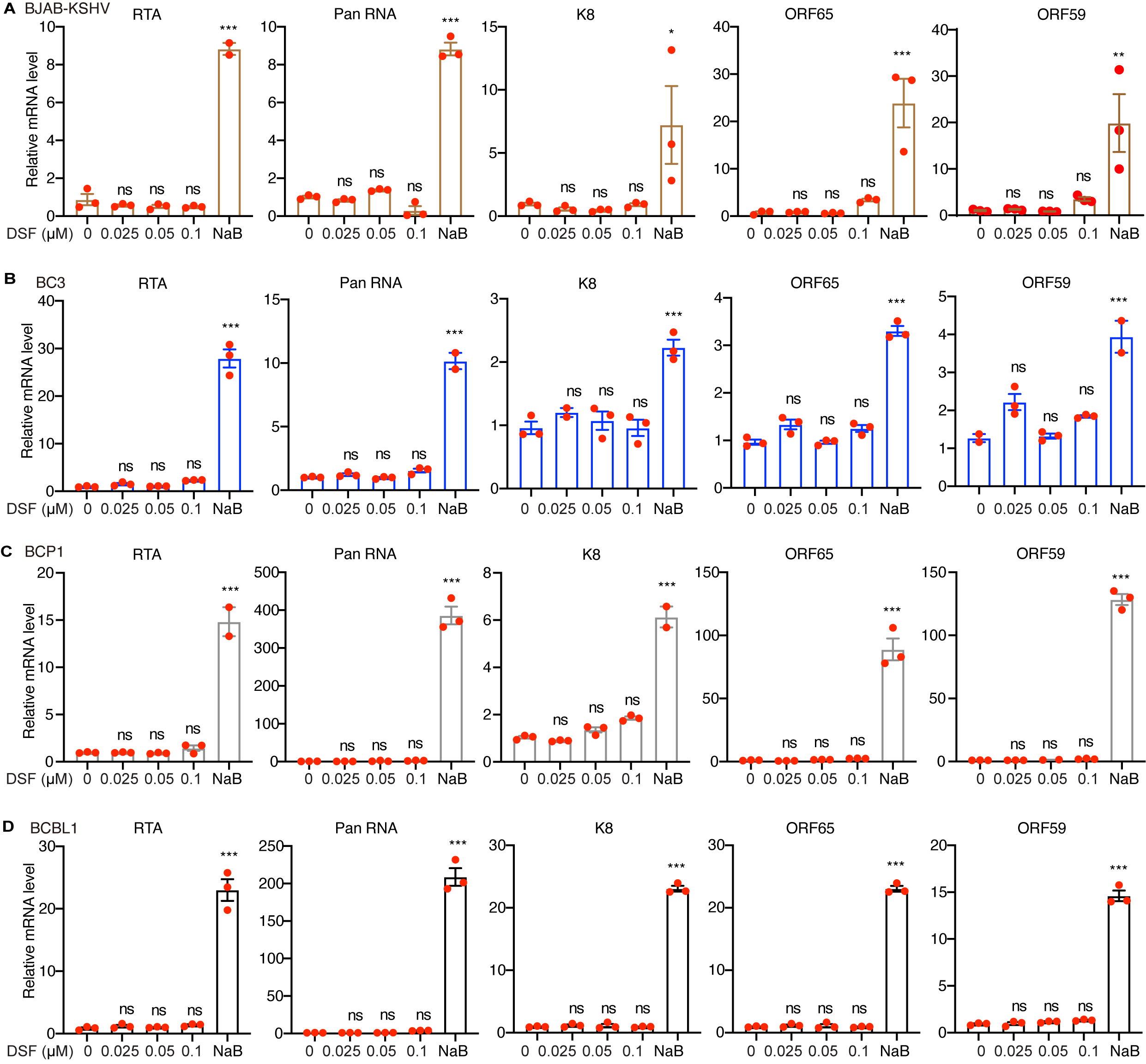

Supplement: S2 Fig — (A) RT-qPCR analysis of the mRNA levels of KSHV RTA, PAN RNA, K8, ORF65 and ORF59 in BJAB-KSHV, BC3, BCP1, and BCBL1 cells treated with 0, 0.025,0.05,0.1 μM DSF or 0.5 mM NaB for 72 h. *, p<0.05, **, p<0.01, ***, p<0.001, ns, not significant compared to 0 μM DSF. (TIF) [file ppat.1012957.s002.tif]

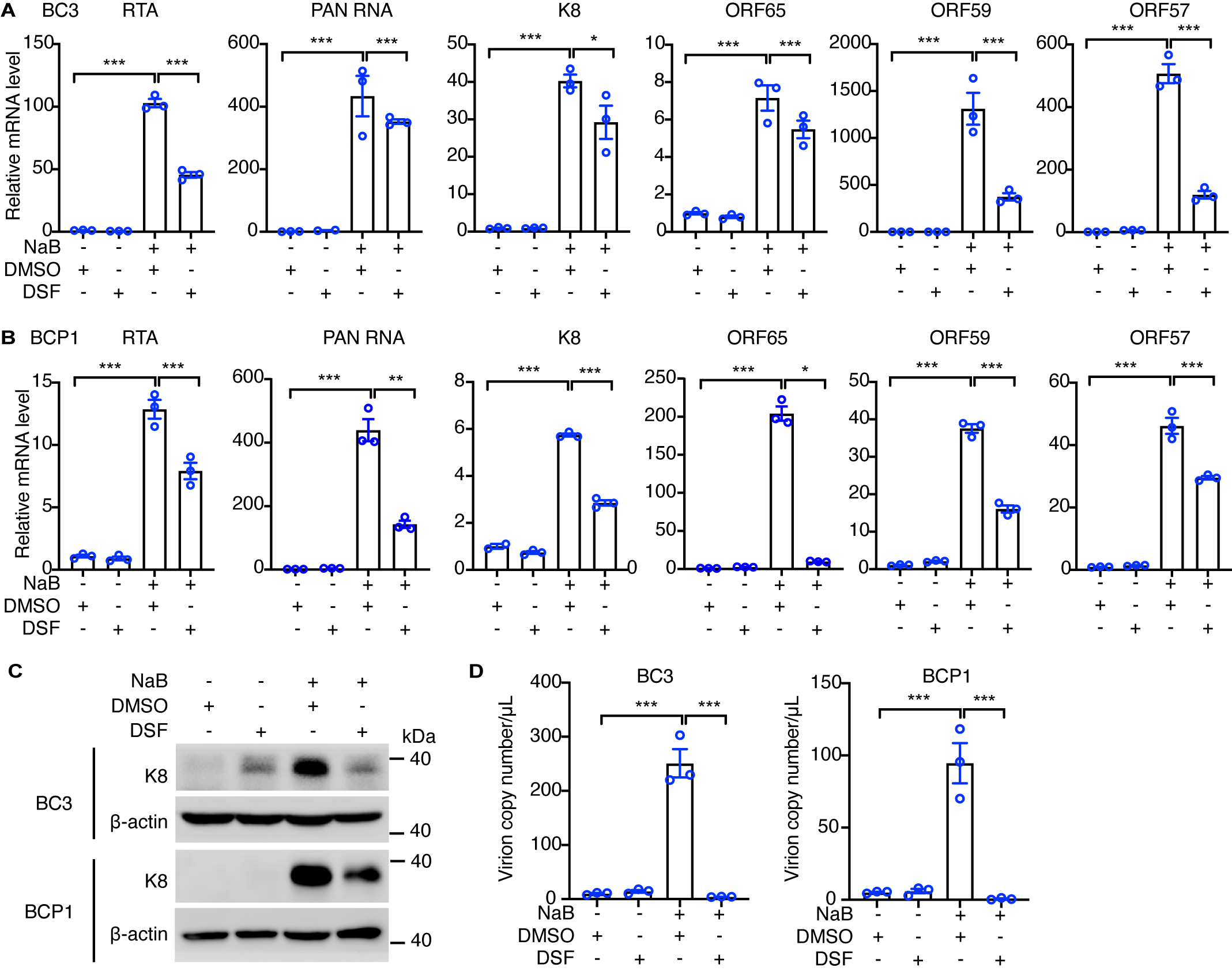

Supplement: S3 Fig — (A-B) RT-qPCR analysis of the mRNA levels of KSHV RTA, PAN RNA, K8, ORF65, ORF59 and ORF57 in BC3 (A) and BCP1 (B) cells treated with 0.1 μM DSF,0.5 mM NaB or both for 72 h. (C) The protein level of K8 was examined by western blots following the treatment of 0.1 μM DSF, 0.5 mM NaB or both for 72 h in BC3 and BCP1 cells. (D)The produced KSHV virions in the supernatants of BC3 and BCP1 cells treated with 0.1 μM DSF, 0.5 mM NaB or both for 96 h was detected by qPCR. *, p<0.05, **, p<0.01, ***, p<0.001, ns, not significant. (TIF) [file ppat.1012957.s003.tif]

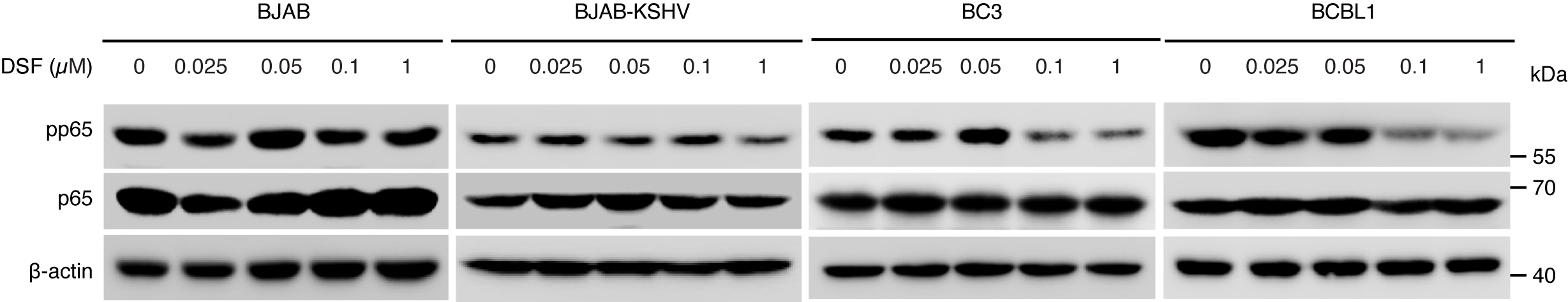

Supplement: S4 Fig — The protein level of the phosphorylation of p65 at S276 and total p65 following the treatment of 1 μM DSF for 24 h in BJAB, BJAB-KSHV, BC3 and BCBL1 cells. (TIF) [file ppat.1012957.s004.tif]

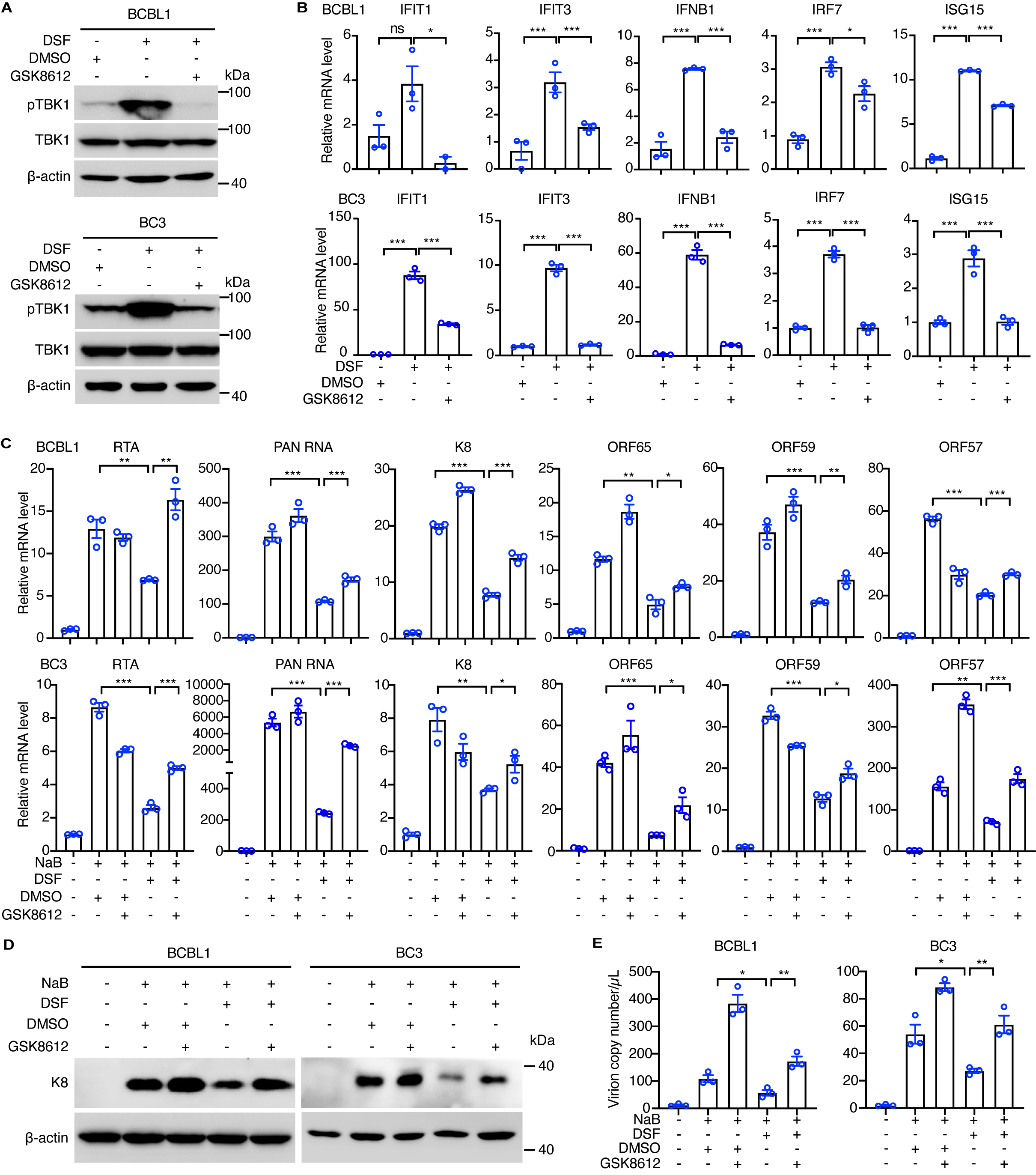

Supplement: S5 Fig — (A) Western blotting analysis of BCBL1 and BC3 cells treated with 0 (DMSO), 1 μM DSF, 0.2 μM GSK8612 or both for 24h. (B) RT-qPCR analysis of the mRNA levels of IFNB1 and ISGs including IFIT1, IFIT3, IRF7, and ISG15 in BCBL1 and BC3 cells treated with 1 μM DSF, 0.2 μM GSK8612 or both for 24 h. (C) RT-qPCR analysis of the mRNA levels of KSHV RTA, PAN RNA, K8, ORF65, ORF59 and ORF57 in BCBL1 and BC3 cells treated with 0.5 mM NaB, 0.1 μM DSF, 0.5 μM GSK8612, both or three of them for 72 h. (D) The protein level of K8 was examined by western blots following the treatment of 0.5 mM NaB, 0.1 μM DSF, 0.5 μM GSK8612, both or three of them in BCBL1 and BC3 cells for 72 h. (E) The produced KSHV virions in the supernatants of BCBL1 and BC3 cells treated with 0.5 mM NaB, 0.1 μM DSF, 0.5 μM GSK8612, both or three of them for 96 h was quantitated by qPCR. *, p<0.05, **, p<0.01, ***, p<0.001, ns, not significant. (TIF) [file ppat.1012957.s005.tif]

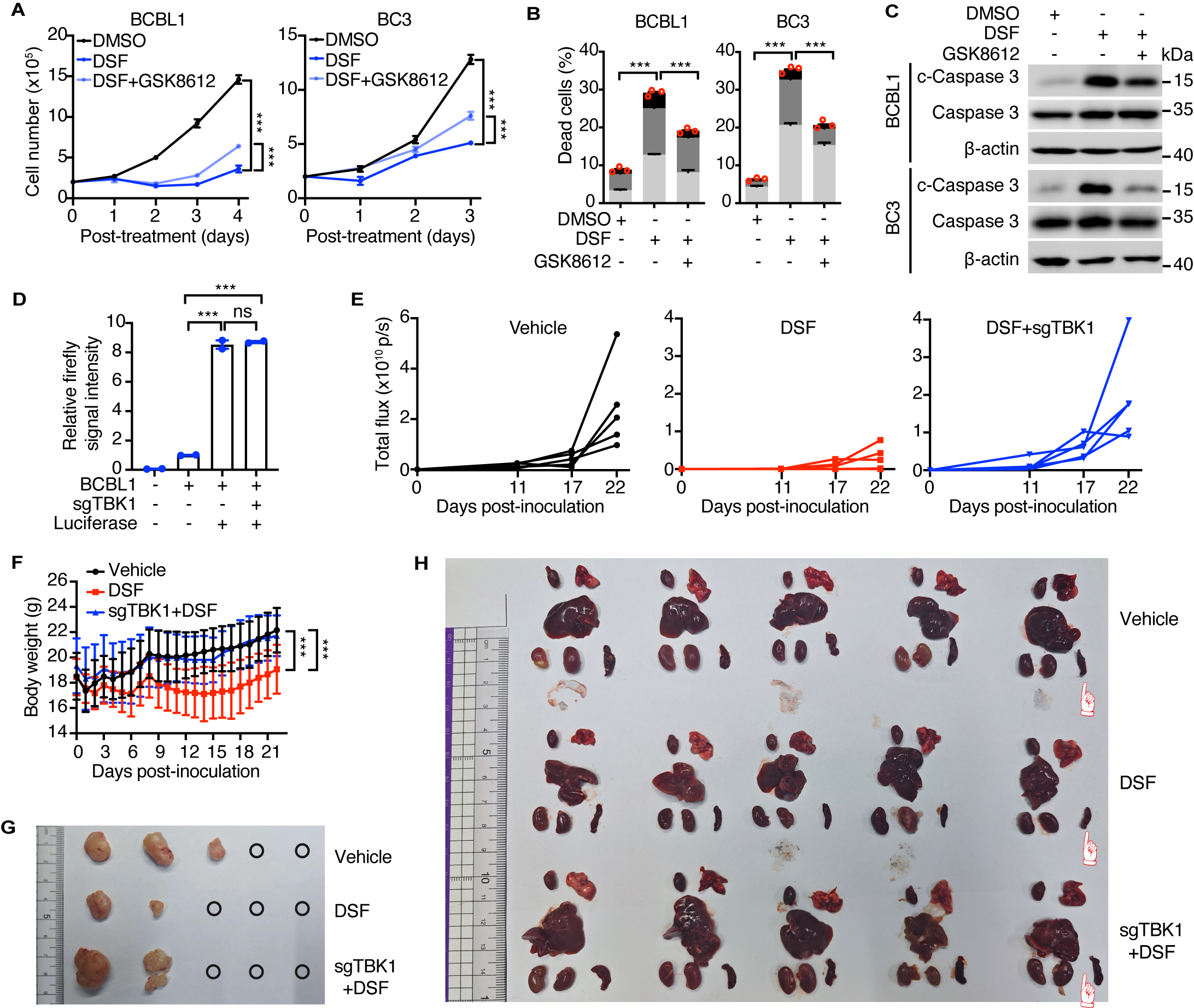

Supplement: S6 Fig — (A) Proliferation curves of BC3 and BCBL1cells treated with 0.1 μM DSF, 0.2 μM GSK8612 or both for continual three days. (B) Apoptosis was detected by flow cytometry with Annexin V and PI staining in BCBL1and BC3 cells treated with 0.1 μM DSF, 0.2 μM GSK8612 or both for three days. (C) Western blotting detection in BCBL1 and BC3 cells treated with 0.1 μM DSF, 0.5 μM GSK8612 or both for three days. (D) Bioluminescence signal of BCBL1-Luc wildtype and TBK1 knockout cells in a 96-well plate. (E) Quantification of luminescence signals from PEL tumors in individual mouse. (F) Body weights of mice. (G) Photograph of solid tumors in the peritoneum and retroperitoneum. (H) Photograph of organs including heart, lung, liver, kidney, pancreas, and spleen of mice engrafted with BCBL1-Luc wildtype or TBK1 knockout cells and treated with vehicle or DSF. (TIF) [file ppat.1012957.s006.tif]
