# Supplementary material for: Repurposing alcohol-abuse drug disulfiram for the treatment of KSHV-infected primary effusion lymphoma by activating antiviral innate immunity
Source: PLoS Pathog. 2025 Mar 4;21(3):e1012957. doi: 10.1371/journal.ppat.1012957 (PMC11922253; doi:10.1371/journal.ppat.1012957)
Supplement: S1 Table — (DOCX) [file ppat.1012957.s007.docx]

**S1 Table. Primer sequences**

| **Primers** | **Sequences (5′ to 3′)** |
| --- | --- |
| **For RT-qPCR analysis** | |
| β-actin-F | TTGCCGACAGGATGCAGAAG |
| β-actin-R | GTACTTGCGCTCAGGAGGAG |
| ORF57-F | AGGTCCCCCTCACCAGTAAA |
| ORF57-R | GAGGACGTGTGTTTTGACCG |
| K8-F | CATGCTGATGCGAATGTGC |
| K8-R | AGCTTCAACATGGTGGGAGTG |
| RTA-F | CACAAAAATGGCGCAAGATGA |
| RTA-R | TGGTAGAGTTGGGCCTTCAGTT |
| ORF65-F | ATATGTCGCAGGCCGAATA |
| ORF65-R | CCACCCATCCTCCTCAGATA |
| ORF59-F | CGAGTCTTCGCAAAAGGTTC |
| ORF59-R | AAGGGACCAACTGGTGTGAG |
| PAN RNA-F | TTTAGCACTGGGACTGCCC |
| PAN RNA-R | CAAGAAGGCAAGCAGCGAG |
| ISG15-F | TGGACAAATGCGACGAACCT |
| ISG15-R | CCTCGAAGGTCAGCCAGAAC |
| IFIT1-F | GCGCTGGGTATGCGATCTC |
| IFIT1-R | CAGCCTGCCTTAGGGGAAG |
| IFNB1-F | CAGCAATTTTCAGTGTCAGAAGC |
| IFNB1-R | TCATCCTGTCCTTGAGGCAGT |
| IFIT3-F | TGAGGAAGGGTGGACACAACTGAA |
| IFIT3-R | AGGAGAATTCTGGGTTGTTGGGCT |
| IRF7-F | TGGTCCTGGTGAAGCTGGAA |
| IRF7-R | GATGTCGTCATAGAGGCTGTTGG |
| IFIT3-F | TGAGGAAGGGTGGACACAACTGAA |
| IFIT3-R | AGGAGAATTCTGGGTTGTTGGGCT |
| **sgRNAs** | |
| sgTBK1-F-1 | CACCGAGAGCACTTCTAATCATCTG |
| sgTBK1-R-1 | AAACCAGATGATTAGAAGTGCTCTC |
| sgTBK1-F-2 | CACCGAATCAAGAACTTATCTACGA |
| sgTBK1-R-2 | AAACTCGTAGATAAGTTCTTGATTC |
| sgIRF3-F | AAACACGCGTGCTGTTCGGAGAGTC |
| sgIRF3-R | AAACGCCGTAGGCCGTGCTTCCAAC |
